# Impact of UK Tobacco Control Policies on Inequalities in Youth Smoking Uptake: A Natural Experiment Study

**DOI:** 10.1093/ntr/ntaa101

**Published:** 2020-05-29

**Authors:** Philip Emeka Anyanwu, Peter Craig, Srinivasa Vittal Katikireddi, Michael James Green

**Affiliations:** 1 MRC/CSO Social and Public Health Sciences Unit, Institute of Health and Wellbeing, University of Glasgow, Glasgow, UK; 2 Global Digital Health Unit, School of Public Health, Imperial College London, London, UK

## Abstract

**Introduction:**

UK countries implemented smoke-free public places legislation and increased the legal age for tobacco purchase from 16 to 18 years between 2006 and 2008. We evaluated the immediate and long-term impacts of these UK policy changes on youth smoking uptake and inequalities therein.

**Aims and Methods:**

We studied 74 960 person-years of longitudinal data from 14 992 youths (aged 11–15 years) in annual UK household surveys between 1994 and 2016. Discrete-time event history analyses examined whether changes in rates of youth smoking transitions (initiation, experimentation, and escalation to daily smoking or quitting) or their inequalities (by parental education) were associated with policy implementation. Parallel analyses examined smoke-free legislation and the change in legal age. We interpret the results as a combined effect of the two pieces of legislation as their implementation dates were too close to identify separate effects. Models were adjusted for sex, age, UK country, historical year, tobacco taxation, and e-cigarette prevalence, with multiple imputation for missing data.

**Results:**

For both policies, smoking initiation reduced following implementation (change in legal age odds ratio [OR]: 0.67; 95% confidence interval [CI]: 0.55 to 0.81; smoke-free legislation OR: 0.68; 95% CI: 0.56 to 0.82), while inequalities in initiation narrowed over subsequent years. The legal age change was associated with annual increases in progression from initiation to occasional smoking (OR: 1.26; 95% CI: 1.07 to 1.50) and a reduction in quitting following implementation (OR: 0.57; 95% CI: 0.35 to 0.94). Similar effects were observed for smoke-free legislation but CIs overlapped the null.

**Conclusions:**

Policies such as these may be highly effective in preventing and reducing socioeconomic inequalities in youth smoking initiation.

**Implications:**

UK implementation of smoke-free legislation and an increase in the legal age for tobacco purchase from 16 to 18 years were associated with an immediate reduction in smoking initiation and a narrowing of inequalities in initiation over subsequent years. While the policies were associated with reductions in the initiation, progression to occasional smoking increased and quitting decreased following the legislation.

## Introduction

Tobacco smoking is reducing globally, but a rapid increase in smoking prevalence is predicted in low- and middle-income countries,^1,2^ and socioeconomic inequalities in smoking are major contributors to inequalities in health in high-income countries.^3–6^ Most smoking begins during adolescence, those who establish an addiction earlier are more likely to smoke into adulthood,^7,8^ and inequalities in youth smoking uptake^9–11^ can be an important driver of inequalities in smoking among adults.^12^ Prevention of youth smoking and reducing inequalities therein are important to achieving a smoke-free generation^13–15^ and reducing the unequal burdens of disease that stem from smoking.

The United Kingdom implemented a comprehensive ban on smoking in public places in 2006 in Scotland^16^ and in 2007 for the rest of the United Kingdom.^17^ Smoke-free legislation has been associated with reductions in cardiovascular and respiratory conditions and pregnancy-related complications among others^18–21^ and may have helped change social norms in the United Kingdom with smoking becoming less acceptable in public places.^22,23^ There is some evidence that smoke-free policies can reduce youth smoking as well.^24–26^ The World Health Organization (WHO) recommends smoke-free public places legislation to promote a smoke-free environment and reduce the harmful effects of secondhand smoke exposure (SHS),^27,28^ especially among nonsmokers.^29^ However, many countries, especially low-middle income countries still do not have such legislation in place.^30^

The minimum legal age for tobacco purchase in the United Kingdom was also raised from 16 to 18 years in 2007 for England, Wales, and Scotland and in 2008 for Northern Ireland,^31,32^ that is, closely coinciding with the implementation of smoke-free policies. There is evidence that this reduced rates of regular smoking in UK youth^33^ and the WHO also recommends restrictions on sales of tobacco products to minors.^27^

While an Australian study demonstrated that adequately funded tobacco control measures could reduce adolescent smoking rates across socioeconomic groups,^34^ equity impacts of policies such as smoke-free legislation or raising the legal age for tobacco purchase are especially important as few tobacco control interventions (except taxation) have as yet been able to demonstrate much success in reducing socioeconomic inequalities in smoking.^9,35^ Equity impacts on youth smoking are often not even examined.^9^ Existing evidence on the change in legal age suggests that impacts were similar across socioeconomic groups,^33^ while a US study found that smoke-free legislation was associated with larger reductions in smoking prevalence among more advantaged young people.^26^

This study aims to estimate the immediate and long-term impacts of these UK tobacco control policies on youth smoking uptake and on socioeconomic inequalities therein. Our study allows for the investigation of both the immediate and long-term impacts of the legislation, and so is the most comprehensive study to date of the equity impacts of these policies on youth smoking uptake.

## Methods

### Study Design

This study followed a pre-published protocol^36^ and complies with the TIDieR-PHP reporting guideline for evaluations of population health and policy interventions.^37^ In line with previous work, smoking uptake was defined as a series of transitions, namely, *initiation* (ie, going from never having tried smoking, to having tried it once or twice); *experimentation* (or progression from having tried smoking once or twice to occasional but less than daily smoking); and *escalation* from occasional to daily use or *quitting* (without escalating to daily smoking).^11^ Of course, further transitions beyond these are possible (eg, quitting after escalating to daily smoking, or relapse after quitting), but our study focuses on these initial stages of uptake. Discrete-time event history analyses were conducted to examine whether changes in the probability of youth smoking transitions were associated with the implementation of the two policies. The implementation of these policies was considered a natural experiment,^38^ and analyses investigated whether there was an immediate or long-term change in the probability of smoking transitions after the country-specific implementation dates of each policy (eg, smoke-free legislation was implemented in 2006 for Scotland and in 2007 for the rest of the United Kingdom). Each of the transitional stages was investigated using a separate model (ie, there was a model for initiation, one for experimentation, and another with escalation and quitting treated as alternate outcomes).

To avoid conflating factors affecting risk for different transitional stages, youths were only considered at risk for a smoking transition once they had made the preceding transition (ie, they were only at risk for experimentation once they had tried smoking once or twice). Consequently, each analysis included all person-years occurring between the ages of 11 and 15 (inclusive) except for those occurring after the transition of interest, or before the previous transition had occurred.

### Data

Data constituted 74 960 person-years for 14 992 youths (each youth contributed data for five observations representing ages 11–15 years) from the British Household Panel Survey (BHPS) Youth Sub-Sample (1994–2008) and its follow-up Understanding Society (2009–2016). Of the total number of persons who completed the last wave of BHPS, 79.4% were followed up in Understanding Society.^39^ Both studies consist of random samples of UK households with booster samples for ethnic minorities (Understanding Society) and Scotland, Wales, and Northern Ireland (BHPS) and include data from youth self-completion questionnaires by young people aged 11–15 years. At age 16 years, participants in the youth subsample became part of the adult main survey and were not included in our analysis from that point.^40^ The rates of nonresponse in both studies are similar to those of other panel studies.^39,41^ The overall proportion of response for the youth self-completion questionnaire in the initial waves of Understanding Society was more than 77%.^39^ The response rate for individual interviews in the first waves of BHPS was more than 85% and this increased in subsequent waves.^41^

### Variables

#### Outcome

The outcomes of interest were the smoking transitional stages. Transitions were coded based on annual self-reports of smoking status (Question: *Do you ever smoke cigarettes at all?* Response categories: *Yes; No)* and frequency (Question: *Please read the statements below and tick the box beside the statement that describes you best.* Response categories: *I have smoked only once or twice; I sometimes smoke but not every week; I usually smoke between one and six cigarettes a week; I usually smoke more than six cigarettes a week; I used to smoke but I don’t now*).^42,43^ Year-by-year histories from age 11 to 15 were created for each respondent. Initiation was coded as a transition from nonsmoking to any of the other smoking frequency categories, experimentation as a transition from having only smoked once or twice to any of the other frequency categories. Escalation and quitting were treated as alternative outcomes (relative to continued occasional smoking), respectively, representing transitions from other states to six or more cigarettes weekly or to saying that they used to smoke but don’t now.^11^ Retrospective data on the age of initiation were used to fill some gaps in prospective smoking histories.

#### Policy Implementation

The primary exposure variables were coded as for an interrupted time series analysis^44^ with a binary indicator that the policy had been implemented and a continuous variable representing the number of years since the implementation of the legislation (representing the change in trend following the legislation). This was computed using country and interview year. Since fieldwork for the BHPS was conducted in the last quarter of each year (with legislation implemented earlier), respondents in Scotland were covered by smoke-free legislation from 2006 and those in the rest of the United Kingdom from 2007. Respondents in England, Wales, and Scotland were covered by the increased legal age for purchase from 2007, with those in Northern Ireland covered from 2008. While our original intention had been to try and separate the effects of these two policies,^36^ this seemed infeasible in practice as, due to the coincident implementation dates, the data only included 698 person-years (<1%) where coverage of the two policies differed. We, therefore, present a parallel analysis of each policy and interpret results as representing the combined impact of both policies.

#### Parental Education

The highest educational attainment of respondents’ parents (no qualification, other qualifications, or degree) indicated socioeconomic position. Educational level has been widely used to indicate the socioeconomic position in epidemiological research given its stability compared to other socioeconomic measures like household income or occupation.^11,26^

#### Confounders

Other factors likely to affect youth smoking behavior were controlled for in analyses. These included age (for initiation; for analyses of subsequent transitions age was represented by two variables indicating the age of and years since the previous transition), gender, and country of residence within the United Kingdom (ie, England, Wales, Scotland, or Northern Ireland; this was coded at the person-level as changes were infrequent and none coincided with implementation dates). We accounted for overall temporal trends in smoking take-up by including the historical year (and a quadratic term to allow for nonlinear trends). Further adjustment was made for relevant temporal trends, namely, annual levels of tobacco taxation (measured as tobacco excise duty rate per 1000 sticks), and rising adult e-cigarette prevalence from 2011 which some fear may renormalize tobacco smoking (coded as 0 for periods before 2011, with annual adult prevalence estimates used from 2011 to 2016),^45,46^ though there is little evidence of this to date.^47^

### Statistical Analysis

Analyses of transition timing require a complete history back to the starting point. A respondent, for example, who was observed from age 12 to 15, still requires data at age 11 to determine whether a transition had already occurred at that age. The complete case analysis, therefore, required discarding 42.7% of the fully observed person-years due to missing data on earlier person-years, losing 47.5% of the unique individuals for whom there were fully observed years of data, and inducing a systematic bias against the inclusion of data at older ages. Imputation of missing data meant this observed data did not need to be discarded and therefore helps minimize bias. Multiple imputation was performed with an unconstrained two-level model of analysis variables in Mplus v8.^48^ This allowed for clustering of person-year level data within persons, effectively utilizing the tendency for observations of smoking status and parental education to correlate within individuals over time. Data on smoking status was missing for 23 085 (30.8%) person-years, and 740 youths (4.9%) had no data on parental education, while all other variables were either fully observed or could be derived from the historical year. Imputations of smoking status and parental education were informed by all analysis variables and by selected auxiliary variables (parental occupational class, parental smoking, and household income). Smoking status was imputed as an ordered categorical variable (nonsmoker, ex-smoker, occasional smoker, and daily smoker), before being recoded into smoking transition variables within each imputed dataset. Results were averaged across 20 imputed datasets, and the sample characteristics of the imputed sample did not differ considerably from those of the fully observed person-years.

The discrete-time event history models for each of the transitional stages were run with smoke-free legislation as the exposure and then repeated replacing smoke-free legislation with the change in legal age (we also include a mutually adjusted model as supplementary information, but this is difficult to interpret with such high collinearity). All models included the policy implementation variables, to indicate the immediate and long-term impacts of the policy change, and parental education to estimate inequalities in transition probabilities. Interactions between parental education and years since the smoke-free legislation implementation were included to investigate the long-term equity impact of the legislation. Each model was also adjusted for sex, age, UK country, historical year, tobacco taxation, and national adult e-cigarette prevalence. Results are presented as odds ratios (ORs) with 95% confidence intervals (95% CIs). Initiation and experimentation were modeled using logistic regression while quitting and escalation were modeled as alternate outcomes using multinomial logistic regression (with continued occasional smoking as the reference category). Predicted probabilities were calculated from the legal age change models in order to demonstrate trends, but findings from the smoke-free legislation models looked very similar. Probability calculations used arbitrary values for variables that were not of interest, and probability calculations from the multinomial model of quitting and escalation incorporate both outcomes such that a change in the risk for one outcome can affect predicted probabilities for the other. Both ORs and predicted probabilities are based only on those who are at risk for that transition (ie, those who have made the previous transition but not yet made the one in question). We further examined the sensitivity of findings to an analysis using placebo implementation dates 5 years before and after the actual implementation dates.

## Results

### Descriptive

Sample characteristics are given in Supplementary Table 1, with both policies covering just below 75% of the 74 960 observed person-years. Supplementary Table 2 presents some descriptive data on the timing of smoking transitions. Approximately one-third of the 14 992 youths in the study had tried smoking by age 15, with a mean initiation age of 13.7 years. Seventy-three percent of these progressed to occasional smoking, the majority of whom then either escalated to daily smoking (28%) or quit before doing so (61%). When examining complete cases, transitions appeared to occur earlier and less frequently, but this is likely due to the systematic bias against the inclusion of data from later person-years.

### Policy Impacts

**Figure 1.**
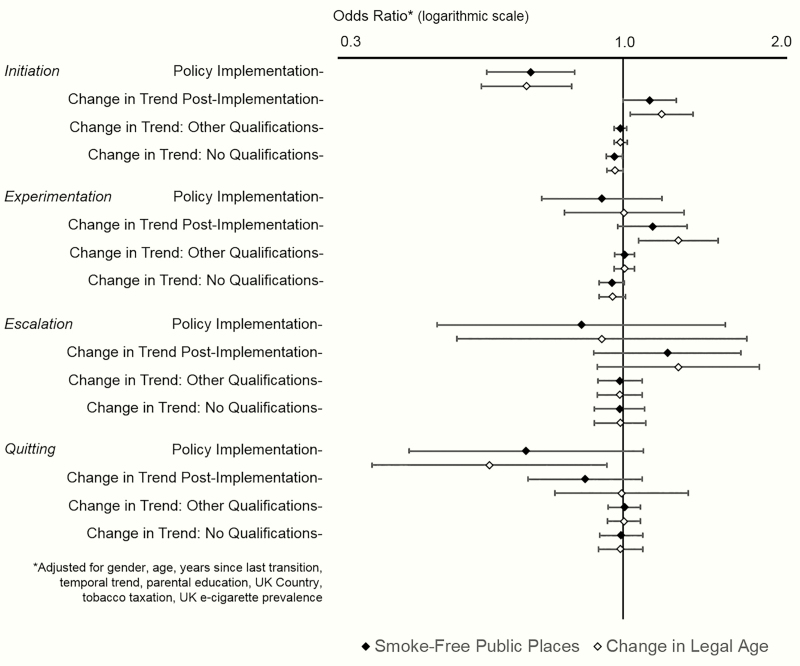
The effect of the policies on smoking transitions.

**Figure 2.**
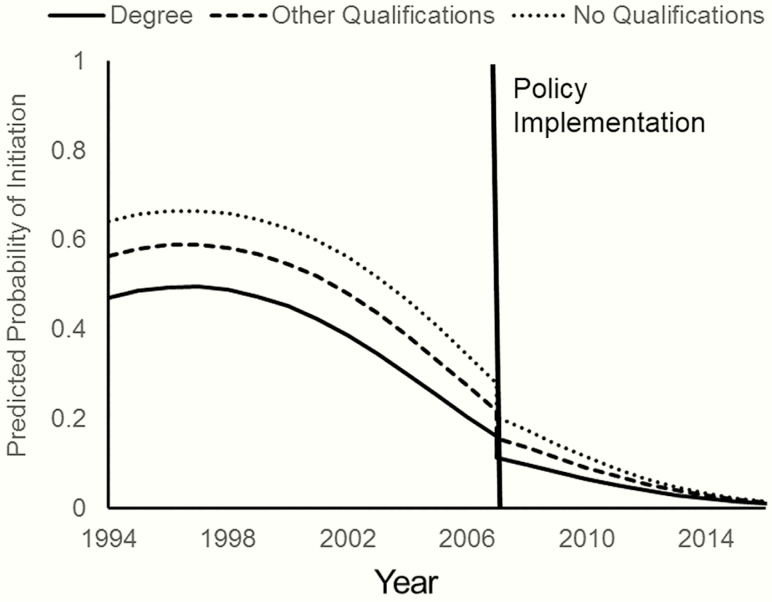
Effect of policy implementation on youth smoking initiation by parental education. Predicted probabilities are calculated based on the change in legal age models from Supplementary Table 3, for age 15 females in England, with e-cigarette prevalence at 0, and the tobacco excise duty rate held constant.

The full details of the models are presented in Supplementary Table 3. Figure 1 shows ORs and 95% CIs representing the immediate impact of each policy on each smoking transition, and the change in trend following implementation (ie, the additional difference in risk per year since implementation). We also present ORs for the interaction between the change in trend and parental education (ie, the additional difference in risk per year since implementation for those whose parents had other or no qualifications relative to degree level education). The clearest evidence for impacts of the two policies is on the risk for initiation, and Figure 2 presents predicted probabilities showing how these effects impact on the overall trends in initiation by parental education. Regardless of which policy was treated as the exposure, there was a clear reduction in risk immediately following implementation. There was a post-implementation change in trend in the opposite direction, meaning that the steep pre-implementation downward trend leveled off somewhat in the years following implementation. Additionally, there was an interaction such that inequalities by parental education narrowed with each year following implementation with risk for initiation in all socioeconomic groups converging near zero at the end of the study period.

**Figure 3.**
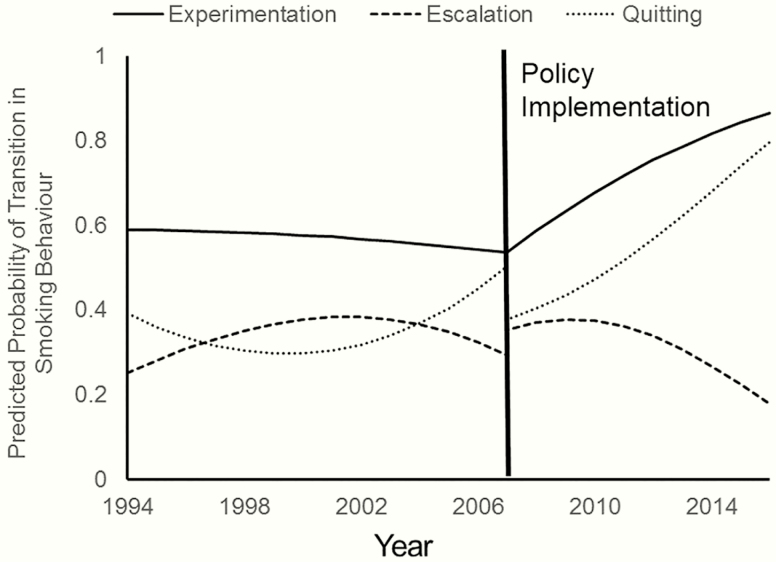
The effect of policy implementation on experimentation, escalation, and quitting. Predicted probabilities are calculated based on the change in legal age models from Supplementary Table 3, for age 15 females in England, with average parental education, e-cigarette prevalence at 0, and the tobacco excise duty rate held constant.

With regard to the postinitiation transitions (experimentation, escalation, and quitting), there was less clear evidence of impacts. Figure 3 presents predicted probabilities showing how the policies impact on the overall trends. The trends for escalation and quitting reflect each other because these were modeled as alternative outcomes; a change in the likelihood of either affects the likelihood of the other, especially since remaining an occasional smoker was relatively rare (the same does not apply to the ORs in Supplementary Table 3 which represent the relative odds of each outcome compared only against the reference category of remaining an occasional smoker). The change in legal age was associated with a change in trend in experimentation such that the odds of progressing to occasional smoking (among those who had initiated) increased in the years following implementation (OR for years since implementation: 1.26; 95% CI: 1.07 to 1.50). The change in legal age was also associated with a reduction in the odds of quitting (OR: 0.57; 95% CI: 0.35 to 0.94), though the pre-implementation trend toward increases in quitting continued in the postimplementation period, with escalation to daily smoking falling accordingly. Similar effects were observed when the smoke-free legislation was treated as the exposure, but these were smaller in magnitude and the CIs included the null (OR: 1.13; 95% CI: 0.98 to 1.31 and OR: 0.66; 95% CI: 0.41 to 1.09, respectively).

Supplementary Table 4 presents a model mutually adjusting for both policies. This indicated that the reduction in initiation associated with policy implementation was most strongly linked to the change in legal age, but for most findings, due to the high collinearity, the effect estimates for the policies diverged and the CIs widened considerably. Supplementary Table 5 presents results from a complete case analysis. The CIs for most estimates overlap with those from the analyses of the imputed data, though there were some differences, for example, the reduction in initiation after policy implementation was not supported, while there was stronger evidence for narrowing of inequalities in initiation and evidence for reductions in experimentation in the years following implementation. These differences are most likely due to biases against the inclusion of data at older ages in the complete case analysis. Sensitivity analyses with placebo implementation dates 5 years before or after actual implementation showed none of the observed impacts on initiation, experimentation, or quitting, though the 5 years after analysis did show an immediate increase in quitting (OR: 2.29; 95% CI: 1.06 to 4.95) with a change in trend in the same direction (OR: 1.48; 95% CIs: 1.07 to 2.03), while the 5 years before analysis showed a change in trend in quitting in the opposite direction (OR: 0.80; 95% CIs: 0.65 to 0.98). Thus, in all cases, the placebo date effects seemed to differ clearly from the “experimental” implementation date effects.

## Discussion

Youth smoking initiation reduced immediately following the implementation of smoke-free public places legislation and an increase in the legal age for tobacco purchase from 16 to 18 years in the United Kingdom. The prelegislation declining trend in initiation flattened off in the years following implementation (perhaps due to a floor effect, as the reduction following implementation meant initiation rates had become very low). There was no immediate equity impact of the legislation, but inequalities in initiation narrowed in the years following the legislation, with rates converging toward zero. Risk of progression to occasional smoking after initiation rose in the years following the legislation and chances of quitting before escalating to daily smoking lowered when the legislation was implemented. These effects on experimentation and quitting were more clearly associated with the change in legal age, similar effects were seen for the smoke-free legislation but they were smaller in magnitude and the CIs crossed the null. However, considering less than 1% of the person-years analyzed differed with respect to whether these policies had been implemented, we are cautious about attributing any of the observed impacts on youth smoking uptake to either policy in isolation of the other. Given the high degree of overlap in coverage of these two policy changes, the impacts observed could represent the effect of either policy or, indeed, the synergistic effect of both policies being implemented together.

The peak levels of initiation we observed in the late 1990s are consistent with our previous work in this dataset, and the subsequent declines in initiation rates roughly coincide with the then Labor government’s increasing focus on tobacco control following their 1998 white paper, “Smoking Kills”.^49^ While many existing studies on the impact of smoke-free legislation on young people concentrate on secondhand smoke exposure,^28^ our study focuses on the immediate and long-term impacts of the two policies introduced across the United Kingdom around 2007 on youth smoking uptake and any inequalities therein. Previous evidence on the implementation of these policies in the United Kingdom has looked primarily at the prevalence of regular smoking in repeat cross-sectional data.^24,33^ Our study is consistent in finding reductions in uptake associated with these policies, but highlights that impacts could be due to either policy (or both) and adds that reductions are concentrated within the initiation stage of uptake, that is, they represent reductions in ever trying smoking. Critically, in contrast with evidence showing limited impacts of smoke-free legislation on inequalities in smoking prevalence among adults,^50^ we observed a narrowing of inequalities in youth initiation following the 2007 legislative changes. This equity impact was not observed in the previous study of the legal age change,^33^ probably because our study had a longer period of follow-up to 2016, while the follow-up to 2008 would not have been able to capture the cumulative narrowing of inequalities that we observed (though they also used eligibility for free school meals to indicate socioeconomic status rather than parental education). Investigations such as this that examine both short- and long-term policy impacts are important because some impacts may take years to accumulate and not be immediately apparent. While we could not clearly distinguish the effects of these two policies, US research which is better able to identify specific effects by capitalizing on cross-state policy variation has shown stronger effects for smoke-free legislation than for restrictions on sales to minors,^25^ though another US study indicated that smoke-free laws could exacerbate rather than alleviate inequalities in youth smoking uptake.^26^

Evidence on the immediate and long-term impacts of smoke-free legislation on quitting among adults is inconsistent. While some studies have reported no difference in quitting after the implementation of smoke-free legislation, other studies have demonstrated a short-lived increase in quitting smoking returning to the prelegislation trend in the years following implementation.^51,52^ Our results suggest, if anything, a reduction in adolescent quitting following the legislation (though we focused on quitting prior to establishing daily smoking) and an increase in progression to occasional smoking after initiation. These impacts may be driven by the reductions in the initiation, with those few who still initiate being those who are most likely to progress to occasional smoking and least likely to quit before establishing daily smoking.

Attribution of our observed impacts as causal effects of the two policies that were introduced rests on the assumption that there were no other confounding factors affecting smoking uptake that also changed at this time, hence we adjusted our analyses for change in the taxation of cigarettes and the rising prevalence of e-cigarettes. We could not distinguish separate effects of each policy due to a high degree of overlap in their coverage, and it is possible that some other contextual change occurred around this time and is responsible for the observed impacts. Furthermore, compliance with both pieces of legislation appears to have been high, secondhand smoke levels in public places and the ease with which young people could purchase cigarettes both fell following implementation,^33,53,54^ and this compliance may have been critical to the effectiveness of the policies in this context. Nevertheless, to the best of our knowledge, our study is the first to provide evidence of the immediate and long-term effects of these UK policies on inequalities in youth smoking uptake.

Regardless of whether it was caused by the smoke-free legislation or the change in legal age, the narrowing of socioeconomic inequalities in initiation in the years since these changes is encouraging, especially given the weak equity impact of other population-level interventions.^35,50^ An area for further research is explicating the mechanisms of these impacts. Where potential mechanisms differ between the two policies, this may help differentiate which policy is actively responsible for the effect. For example, both policies may impact on social norms^22,23^ or restrict access to tobacco,^25,33^ but parental smoking is a known risk factor for youth uptake^55^ and may have been impacted by the smoke-free legislation, but would be unlikely to have been affected by the change in legal age (with most smoking parents aged >18 years).

Implementation of smoke-free legislation and an increase in the legal age for tobacco purchase from 16 to 18 in the United Kingdom were associated with immediate reductions in youth initiation and longer-term reductions in inequalities therein. It was unclear which policy was responsible for this impact; it may have been either or the synergistic effect of both. Regardless of which policy was responsible, our findings demonstrate that public health policies like these, which have not yet been adopted in many countries, especially low-middle income countries,^30^ may be highly effective in preventing and reducing inequalities in youth smoking initiation.

## Supplementary Material

A Contributorship Form detailing each author’s specific involvement with this content, as well as any supplementary data, is available online at https://academic.oup.com/ntr.

ntaa101_suppl_Supplementary_Table_1

ntaa101_suppl_Supplementary_Table_2

ntaa101_suppl_Supplementary_Table_3

ntaa101_suppl_Supplementary_Table_4

ntaa101_suppl_Supplementary_Table_5

ntaa101_suppl_Supplementary_Taxonomy

## References

[1] Bilano V, Gilmour S, Moffiet T, et al. Global trends and projections for tobacco use, 1990–2025: an analysis of smoking indicators from the WHO Comprehensive Information Systems for Tobacco Control. Lancet. 2015;385(9972):966–976. 25784347 10.1016/S0140-6736(15)60264-1

[2] Fitzmaurice C, Akinyemiju TF, Lami FHA, et al. Global, regional, and national cancer incidence, mortality, years of life lost, years lived with disability, and disability-adjusted life-years for 29 cancer groups, 1990 to 2016: a systematic analysis for the global burden of disease study. JAMA Oncol. 2018;4(11):1553–1568. doi:10.1001/jamaoncol.2018.2706 29860482 10.1001/jamaoncol.2018.2706 PMC6248091

[3] Reid JL, Hammond D, Driezen P Socio-economic status and smoking in Canada, 1999–2006: has there been any progress on disparities in tobacco use? Can J Public Heal Can Santee Publique. Published online 2010;101(1):73–78. 10.1007/BF03405567 PMC6973977 20364543

[4] Siahpush M, English D, Powles J The contribution of smoking to socioeconomic differentials in mortality: results from the Melbourne Collaborative Cohort Study, Australia. J Epidemiol Community Health. 2006;60(12):1077–1079. 17108305 10.1136/jech.2005.042572 PMC2465498

[5] Jha P, Peto R, Zatonski W, Boreham J, Jarvis MJ, Lopez AD Social inequalities in male mortality, and in male mortality from smoking: indirect estimation from national death rates in England and Wales, Poland, and North America. Lancet. 2006;368(9533):367–370. 16876664 10.1016/S0140-6736(06)68975-7

[6] Whitley E, Batty GD, Hunt K, Popham F, Benzeval M The role of health behaviours across the life course in the socioeconomic patterning of all-cause mortality: the west of Scotland twenty-07 prospective cohort study. Ann Behav Med. 2014;47(2):148–157. 24072618 10.1007/s12160-013-9539-x PMC3964290

[7] Wellman RJ, DiFranza JR, Savageau JA, Dussault GF Short term patterns of early smoking acquisition. Tob Control. 2004;13(3):251–257. 15333880 10.1136/tc.2003.005595 PMC1747885

[8] Chassin L, Presson CC, Pitts SC, Sherman SJ The natural history of cigarette smoking from adolescence to adulthood in a midwestern community sample: multiple trajectories and their psychosocial correlates. Health Psychol. 2000;19(3):223–231. 10868766

[9] Brown T, Platt S, Amos A Equity impact of interventions and policies to reduce smoking in youth: systematic review. Tob Control. 2014;23(e2):e98–e105. 24842855 10.1136/tobaccocontrol-2013-051451

[10] Barreto SM, de Figueiredo RC, Giatti L Socioeconomic inequalities in youth smoking in Brazil. BMJ Open. 2013;3(12):e003538. 10.1136/bmjopen-2013-003538 PMC3855598 24302501

[11] Green MJ, Leyland AH, Sweeting H, Benzeval M Socioeconomic position and early adolescent smoking development: evidence from the British Youth Panel Survey (1994–2008). Tob Control. 2016;25(2):203–210. 25380762 10.1136/tobaccocontrol-2014-051630 PMC4789819

[12] Maralani V Educational inequalities in smoking: the role of initiation versus quitting. Soc Sci Med. 2013;84(1):129–137. 23466258 10.1016/j.socscimed.2013.01.007

[13] The Scottish Government. Creating a Tobacco-Free Generation: a Tobacco Control Strategy for Scotland—Google Search Published online 2013. Accessed October 25, 2017 http://www.gov.scot/resource/0041/00417331.pdf

[14] Reitsma MB, Fullman N, Ng M, et al. Smoking prevalence and attributable disease burden in 195 countries and territories, 1990–2015: a systematic analysis from the Global Burden of Disease Study 2015. The Lancet. 2017;389(10082):1885–1906. doi:10.1016/S0140-6736(17)30819-X. PMC5439023 28390697

[15] Department of Health. Towards a Smoke-Free Generation: Tobacco Control Plan for England Published 2017 Accessed October 25, 2017 https://www.gov.uk/government/publications/towards-a-smoke-free-generation-tobacco-control-plan-for-england

[16] Scottish Executive. Smoking, Health and Social Care (Scotland) Act 2005 Published 2005 Accessed June 4, 2018 https://www.legislation.gov.uk/asp/2005/13/contents

[17] Department of Health. Health Act 2006. Published 2006 Accessed June 4, 2018 https://www.legislation.gov.uk/ukpga/2006/28/contents

[18] Mackay D, Haw S, Ayres JG, Fischbacher C, Pell JP Smoke-free legislation and hospitalizations for childhood asthma. N Engl J Med. 2010;363(12):1139–1145. 20843248 10.1056/NEJMoa1002861

[19] Faber T, Kumar A, Mackenbach JP, et al. Effect of tobacco control policies on perinatal and child health: a systematic review and meta-analysis. Lancet Public Health. 2017;2(9):e420–e437. 28944313 10.1016/S2468-2667(17)30144-5 PMC5592249

[20] Been JV, Nurmatov UB, Cox B, Nawrot TS, van Schayck CP, Sheikh A Effect of smoke-free legislation on perinatal and child health: a systematic review and meta-analysis. Lancet. 2014;383(9928):1549–1560. 24680633 10.1016/S0140-6736(14)60082-9

[21] Frazer K, Callinan JE, McHugh J, et al. Legislative smoking bans for reducing harms from secondhand smoke exposure, smoking prevalence and tobacco consumption. Cochrane Database Syst Rev. 2016;2(2):CD005992. 26842828 10.1002/14651858.CD005992.pub3 PMC6486282

[22] Ritchie D, Amos A, Martin C Public places after smoke-free—a qualitative exploration of the changes in smoking behaviour. Health Place. 2010;16(3):461–469. 20044297 10.1016/j.healthplace.2009.12.003

[23] Phillips R, Amos A, Ritchie D, Cunningham-Burley S, Martin C Smoking in the home after the smoke-free legislation in Scotland: qualitative study. BMJ. 2007;335(7619):553. 17827488 10.1136/bmj.39301.497593.55 PMC1976533

[24] Katikireddi SV, Der G, Roberts C, Haw S Has childhood smoking reduced following smoke-free public places legislation? A segmented regression analysis of cross-sectional UK school-based surveys. Nicotine Tob Res. 2016;18(7):1670–1674. 26911840 10.1093/ntr/ntw018 PMC4902887

[25] Farrelly MC, Loomis BR, Han B, et al. A comprehensive examination of the influence of state tobacco control programs and policies on youth smoking. Am J Public Health. 2013;103(3):549–555. 23327252 10.2105/AJPH.2012.300948 PMC3673505

[26] Tauras JA, Huang J, Chaloupka FJ Differential impact of tobacco control policies on youth sub-populations. Int J Environ Res Public Health. 2013;10(9):4306–4322. 24036487 10.3390/ijerph10094306 PMC3799499

[27] World Health Organization. WHO Report on the Global Tobacco Epidemic, 2017: Monitoring Tobacco Use and Prevention Policies WHO; 2017 http://apps.who.int/iris/bitstream/10665/255874/1/9789241512824-eng.pdf?ua=1&ua=1

[28] Moore GF, Currie D, Gilmore G, Holliday JC, Moore L Socioeconomic inequalities in childhood exposure to secondhand smoke before and after smoke-free legislation in three UK countries. J Public Health (Oxf). 2012;34(4):599–608. 22448041 10.1093/pubmed/fds025 PMC3503469

[29] Britton J Smoke-free policy and child health. Lancet Public Health. 2017;2(9):e392–e393. 29253406 10.1016/S2468-2667(17)30169-X

[30] Uang R, Hiilamo H, Glantz SA Accelerated adoption of smoke-free laws after ratification of the World Health Organization framework convention on tobacco control. Am J Public Health. 2016;106(1):166–171. 26562125 10.2105/AJPH.2015.302872 PMC4689638

[31] Department of Health. The Children and Young Persons (Sale of Tobacco etc.) Order 2007 Published online 2007 Accessed June 20, 2018 http://www.legislation.gov.uk/uksi/2007/767/contents/made

[32] Department of Health. The Children and Young Persons (Sale of Tobacco etc.) Regulations (Northern Ireland) 2008 Published online 2008 Accessed June 20, 2018 http://www.legislation.gov.uk/nisr/2008/306/contents/made

[33] Millett C, Lee JT, Gibbons DC, Glantz SA Increasing the age for the legal purchase of tobacco in England: impacts on socio-economic disparities in youth smoking. Thorax. 2011;66(10):862–865. doi:10.1136/hx.2010.154963. 21502102 10.1136/thx.2010.154963 PMC3158837

[34] White VM, Hayman J, Hill DJ Can population-based tobacco-control policies change smoking behaviors of adolescents from all socio-economic groups? Findings from Australia: 1987–2005. Cancer Causes Control. 2008;19(6):631–640. 18264783 10.1007/s10552-008-9127-8

[35] Hill S, Amos A, Clifford D, Platt S Impact of tobacco control interventions on socioeconomic inequalities in smoking: review of the evidence. Tob Control. 2014;23(e2):e89–e97. 24046211 10.1136/tobaccocontrol-2013-051110

[36] Anyanwu PE, Craig P, Katikireddi SV, Green MJ Impacts of smoke-free public places legislation on inequalities in youth smoking uptake: study protocol for a secondary analysis of UK survey data. BMJ Open. 2018;8(3):e022490. 10.1136/bmjopen-2018-022490 PMC5875608 29593026

[37] Campbell M, Katikireddi SV, Hoffmann T, Armstrong R, Waters E, Craig P TIDieR-PHP: a reporting guideline for population health and policy interventions. BMJ. 2018;361:k1079. 29769210 10.1136/bmj.k1079 PMC5954974

[38] Craig P, Katikireddi SV, Leyland A, Popham F Natural experiments: an overview of methods, approaches, and contributions to public health intervention research. Annu Rev Public Health. 2017;38:39–56. 28125392 10.1146/annurev-publhealth-031816-044327 PMC6485604

[39] Lynn P, Burton J, Kaminska O, Knies G, Nandi A An initial look at non-response and attrition in Understanding Society. Underst Soc Work Pap Ser. 2012;2.

[40] University of Essex. Institute for Social and Economic Research, NatCen, Kantar Public. Understanding Society: Waves 1–9, 2009–2018 and Harmonised BHPS: Waves 1–18, 1991–2009 Published online 2019. Accessed November 20, 2019. https://www.understandingsociety.ac.uk/sites/default/files/downloads/documentation/mainstage/user-guides/mainstage-user-guide.pdf

[41] Uhrig SC The nature and causes of attrition in the British Household Panel Study. ISER Working Paper Series. 2008.

[42] University of Essex. Institute for Social and Economic Research, NatCen, Kantar Public. Understanding Society: Young People Self-completion Questionnaire Published online 2019. Accessed November 20, 2019. https://www.understandingsociety.ac.uk/sites/default/files/downloads/documentation/mainstage/questionnaire/wave-10/W10-gb-youth-self-completion-questionnaire.pdf

[43] University of Essex, Economic and Social Research Council. BHPS Questionnaires and Survey Documents Published online 2000 https://www.iser.essex.ac.uk/bhps/documentation/pdf_versions/survey_docs/wave6/index.html

[44] Bernal JL, Cummins S, Gasparrini A Interrupted time series regression for the evaluation of public health interventions: a tutorial. Int J Epidemiol. 2017;46(1):348–355. 27283160 10.1093/ije/dyw098 PMC5407170

[45] Action on Smoking and Health. Use of Electronic Cigarettes (Vapourisers) Among Adults in Great Britain Published 2016 Accessed November 22, 2017 http://colinmendelsohn.com.au/files/2814/9446/2312/ASH._Use_of_electronic_cigarettes_vapourisers_among_adults_in_Great_Britain._Fact_sheet._May_2017.pdf

[46] Office for National Statistics. E-cigarette Use in Great Britain Published 2017 Accessed June 14, 2018 https://www.ons.gov.uk/peoplepopulationandcommunity/healthandsocialcare/drugusealcoholandsmoking/datasets/ecigaretteuseingreatbritain

[47] Hallingberg B, Maynard OM, Bauld L, et al. Have e-cigarettes renormalised or displaced youth smoking? Results of a segmented regression analysis of repeated cross sectional survey data in England, Scotland and Wales. Tob Control. 2019;29(2):207–216. doi:10.1136/tobaccocontrol-2018-054584. 30936390 10.1136/tobaccocontrol-2018-054584 PMC7036293

[48] Muthén LK, Muthén BO. Mplus User’s Guide. CzeglédiE Body Dissatisfaction Trait Anxiety Self-Esteem Young Men. Los Angeles, CA: Muthen & Muthen; 1998–2010. Published online 2016.

[49] UK Secretary of State for Health. Smoking Kills: A White Paper on Tobacco UK Published online 1998 Accessed July 25, 2018 https://www.google.com/search?source=hp&ei=XEBYW4ObL5D4kwWWuY3ICg&q=Smoking+Kills%3A+A+White+Paper+on+Tobacco.+United+Kingdom&oq=Smoking+Kills%3A+A+White+Paper+on+Tobacco.+United+Kingdom&gs_l=psy-ab.3...743.743.0.960.2.1.0.0.0.0.143.143.0j1.1.0....0...1c.1.64.psy-ab..1.0.0.0...0.Hzw6adNpBFc

[50] Thomas S, Fayter D, Misso K, et al. Population tobacco control interventions and their effects on social inequalities in smoking: systematic review. Tob Control. 2008;17(4):230–237. 18426867 10.1136/tc.2007.023911 PMC2565568

[51] Hackshaw L, McEwen A, West R, Bauld L Quit attempts in response to smoke-free legislation in England. Tob Control. 2010;19(2):160–164. 20378592 10.1136/tc.2009.032656

[52] Federico B, Mackenbach JP, Eikemo TA, Kunst AE Impact of the 2005 smoke-free policy in Italy on prevalence, cessation and intensity of smoking in the overall population and by educational group. Addiction. 2012;107(9):1677–1686. 22360495 10.1111/j.1360-0443.2012.03853.x

[53] Semple S, Creely KS, Naji A, Miller BG, Ayres JG Secondhand smoke levels in Scottish pubs: the effect of smoke-free legislation. Tob Control. 2007;16(2):127–132. 17400951 10.1136/tc.2006.018119 PMC2598470

[54] Local Government Analysis and Research. Smoke-Free Legislation Compliance Data Report Covering July–December 2008 (Period 10) Published online 2009. Accessed November 20, 2019. http://www.smokefreeengland.co.uk/files/tob01_01_smokefree_report_period_10_v2.pdf

[55] Tyas SL, Pederson LL Psychosocial factors related to adolescent smoking: a critical review of the literature. Tob Control. 1998;7(4):409–420. 10093176 10.1136/tc.7.4.409 PMC1751465

