# Supplementary material for: Impact of UK Tobacco Control Policies on Inequalities in Youth Smoking Uptake: A Natural Experiment Study
Source: Nicotine Tob Res. 2020 May 29;22(11):1973–80. doi: 10.1093/ntr/ntaa101 (PMC7593354; doi:10.1093/ntr/ntaa101)
Supplement: ntaa101_suppl_Supplementary_Table_1 [file ntaa101_suppl_supplementary_table_1.docx]

**Supplementary Table 1: Sample Characteristics**

|  | | | Imputed Data*  N (%) | Complete Person-Years  N (%) |
| --- | --- | --- | --- | --- |
| Person-level  Imputed  N=14,992  Complete Person-Years**  N=13,634 | Gender | Male | 7,596 (50.7) | 6,913 (50.7) |
|  |  | Female | 7,396 (49.3) | 6,721 (49.3) |
|  | UK Country | England | 11,065 (73.8) | 10,194 (74.8) |
|  |  | Scotland | 1,581 (10.6) | 1,420 (10.4) |
|  |  | Wales | 1,307 (8.7) | 1,181 (8.7) |
|  |  | Northern Ireland | 1,039 (6.9) | 839 (6.2) |
| Person-year level  Imputed  N=74,960  Complete Person-Years  N=35,764 | Age | 11 | 14,992 (20.0) | 7,149 (20.0) |
|  |  | 12 | 14,992 (20.0) | 7,269 (20.3) |
|  |  | 13 | 14,992 (20.0) | 7,304 (20.4) |
|  |  | 14 | 14,992 (20.0) | 7,204 (20.1) |
|  |  | 15 | 14,992 (20.0) | 6,838 (19.1) |
|  | Smoke-Free Legislation | Yes | 55,996 (74.7) | 24,843 (69.5) |
|  |  | No | 18,964 (25.3) | 10,921 (30.5) |
|  | Legal Age for Purchase | 18 | 55,298 (73.8) | 24,412 (68.3) |
|  |  | 16 | 19,662 (26.2) | 11,352 (31.7) |
|  | Parental Education | Degree | 19,265 (25.7) | 9,111 (25.5) |
|  |  | Other qualifications | 46,047 (61.4) | 21,995 (61.5) |
|  |  | No qualification | 9,648 (12.9) | 4,658 (13.0) |
| **Results are averaged across 20 imputed datasets.*  ***Complete Person-Years is based on respondents for whom we had complete data on all analysis variables within a given year, so this figure means we had at least one complete year of data for 13,634 respondents.* | | | | |
