# Supplementary material for: Impact of UK Tobacco Control Policies on Inequalities in Youth Smoking Uptake: A Natural Experiment Study
Source: Nicotine Tob Res. 2020 May 29;22(11):1973–80. doi: 10.1093/ntr/ntaa101 (PMC7593354; doi:10.1093/ntr/ntaa101)
Supplement: ntaa101_suppl_Supplementary_Table_2 [file ntaa101_suppl_supplementary_table_2.docx]

**Supplementary Table 2: Description of smoking transitions**

| Imputed Data* | Initiation | Experimentation | Escalation | Quitting | |
| --- | --- | --- | --- | --- | --- |
| Total Number of Eligible Youth  N (% of all Youth) | 14,992 (100.0) | 5,265 (35.1) | 3,838 (25.6) | 3,838 (25.6) | |
| Transitions by Age 15 years  N (% of Eligible Youth) | 5,265 (35.1) | 3,838 (72.9) | 1,062 (27.7) | 2,329 (60.7) | |
| Average Age at Transition (in years) | 13.7 | 13.9 | 14.2 | 13.9 | |
| Average Number of Years since Preceding Transition | - | 0.5 | 0.2 | 0.2 | |
| **Results are averaged across 20 imputed datasets.* | | | | | |
| *Complete person-years*** | | | | | |
|  | **Initiation** | **Experimentation** | **Escalation** | | **Quitting** |
| Total Number of Eligible Youth  N (% of all Youth) | 7,149 (100.0) | 1,371 (19.2) | 793 (11.1) | | 793 (11.1) |
| Transitions by Age 15 years  N (% of Eligible Youth) | 1,371 (19.2) | 793 (57.8) | 238 (30.0) | | 393 (49.6) |
| Average Age at Transition (in years) | 12.8 | 13.2 | 13.7 | | 13.1 |
| Average Number of Years since Preceding Transition | - | 0.6 | 0.3 | | 0.3 |
| **This sample is considerably smaller than in Supplementary Table 1 because examining transition timing requires a complete history on all earlier person-years. Thus, 15,276 of the fully-observed person-years in Table 1 (42.7%) had to be discarded because there was missing data on earlier person-years. This reduced the sample to 7,149 individuals, losing 6,485 individuals (47.5%) who did have some fully-observed person-years. | | | | | |
