# Supplementary material for: Impact of UK Tobacco Control Policies on Inequalities in Youth Smoking Uptake: A Natural Experiment Study
Source: Nicotine Tob Res. 2020 May 29;22(11):1973–80. doi: 10.1093/ntr/ntaa101 (PMC7593354; doi:10.1093/ntr/ntaa101)
Supplement: ntaa101_suppl_Supplementary_Table_3 [file ntaa101_suppl_supplementary_table_3.docx]

**Supplementary Table 3: Odds Ratios Associated with Smoking Transitions**

|  | **Smoke-Free Legislation** | | **Change in Legal Age for Purchase** | |
| --- | --- | --- | --- | --- |
|  | OR | 95% CIs | OR | 95% CIs |
| *Initiation (ref: Never Smoked)* |  |  |  |  |
| Policy Implementation | 0.68 | 0.56-0.82 | 0.67 | 0.55-0.81 |
| Years Since Implementation | 1.12 | 1.00-1.25 | 1.18 | 1.03-1.34 |
| Historical Year | 0.74 | 0.70-0.78 | 0.73 | 0.69-0.77 |
| Historical Year^2^ | 0.99 | 0.98-0.99 | 0.98 | 0.98-0.99 |
| Other Qualifications (ref: degree) | 1.47 | 1.29-1.67 | 1.46 | 1.29-1.66 |
| No Qualifications (ref: degree) | 2.04 | 1.73-2.42 | 2.03 | 1.71-2.40 |
| Other Qualifications*Years Since Implementation | 0.99 | 0.96-1.02 | 0.99 | 0.96-1.02 |
| No Qualifications*Years Since Implementation | 0.97 | 0.93-1.00 | 0.97 | 0.93-1.00 |
| Tobacco Taxation | 1.02 | 1.02-1.03 | 1.02 | 1.01-1.03 |
| Adult E-cigarette Prevalence | 1.08 | 1.02-1.14 | 1.06 | 1.00-1.13 |
| Male (ref: Female) | 0.93 | 0.87-1.00 | 0.93 | 0.87-1.00 |
| Age | 1.89 | 1.84-1.94 | 1.89 | 1.83-1.94 |
| Wales (ref: England) | 1.06 | 0.94-1.19 | 1.07 | 0.95-1.20 |
| Scotland (ref: England) | 1.04 | 0.92-1.17 | 1.08 | 0.97-1.19 |
| Northern Ireland (ref: England) | 1.02 | 0.89-1.17 | 1.10 | 0.93-1.31 |
| *Experimentation (ref: Initiated only)* |  |  |  |  |
| Policy Implementation | 0.91 | 0.71-1.18 | 1.01 | 0.78-1.29 |
| Years Since Implementation | 1.13 | 0.98-1.31 | 1.26 | 1.07-1.50 |
| Historical Year | 1.01 | 0.94-1.07 | 0.97 | 0.91-1.04 |
| Historical Year^2^ | 1.00 | 1.00-1.01 | 1.00 | 0.99-1.00 |
| Other Qualifications (ref: degree) | 1.02 | 0.83-1.24 | 1.01 | 0.83-1.23 |
| No Qualifications (ref: degree) | 1.58 | 1.23-2.04 | 1.55 | 1.21-2.00 |
| Other Qualifications*Years Since Implementation | 1.01 | 0.96-1.05 | 1.01 | 0.96-1.05 |
| No Qualifications*Years Since Implementation | 0.95 | 0.90-1.01 | 0.96 | 0.91-1.01 |
| Tobacco Taxation | 0.99 | 0.98-1.00 | 0.99 | 0.98-1.00 |
| Adult E-cigarette Prevalence | 0.95 | 0.87-1.04 | 0.95 | 0.87-1.04 |
| Male (ref: Female) | 0.93 | 0.84-1.02 | 0.93 | 0.84-1.02 |
| Age at Initiation | 1.17 | 1.12-1.23 | 1.17 | 1.12-1.23 |
| Years Since Initiation | 1.31 | 1.22-1.42 | 1.32 | 1.22-1.43 |
| Wales (ref: England) | 0.93 | 0.79-1.10 | 0.94 | 0.80-1.11 |
| Scotland (ref: England) | 0.87 | 0.74-1.02 | 0.92 | 0.79-1.07 |
| Northern Ireland (ref: England) | 0.88 | 0.71-1.08 | 1.03 | 0.80-1.32 |
| *Escalation to Daily Smoking (ref: Occasional Smoking)* |  |  |  |  |
| Policy Implementation | 0.84 | 0.46-1.54 | 0.92 | 0.50-1.69 |
| Years Since Implementation | 1.21 | 0.89-1.64 | 1.26 | 0.90-1.78 |
| Historical Year | 1.02 | 0.87-1.64 | 1.00 | 0.86-1.17 |
| Historical Year^2^ | 1.00 | 0.99-1.01 | 1.00 | 0.98-1.01 |
| Other Qualifications (ref: degree) | 1.44 | 1.00-2.09 | 1.45 | 1.00-2.09 |
| No Qualifications (ref: degree) | 1.90 | 1.20-3.03 | 1.88 | 1.19-2.99 |
| Other Qualifications*Years Since Implementation | 0.99 | 0.90-1.08 | 0.99 | 0.90-1.08 |
| No Qualifications*Years Since Implementation | 0.99 | 0.89-1.10 | 0.99 | 0.89-1.10 |
| Tobacco Taxation | 0.99 | 0.97-1.01 | 0.99 | 0.97-1.01 |
| Adult E-cigarette Prevalence | 0.83 | 0.68-1.01 | 0.84 | 0.68-1.02 |
| Male (ref: Female) | 0.79 | 0.65-0.96 | 0.79 | 0.65-0.96 |
| Age at Experimentation | 1.29 | 1.18-1.42 | 1.29 | 1.18-1.42 |
| Years of Experimentation | 2.08 | 1.57-2.75 | 2.08 | 1.57-2.76 |
| Wales (ref: England) | 1.19 | 0.85-1.69 | 1.20 | 0.85-1.70 |
| Scotland (ref: England) | 0.97 | 0.69-1.36 | 1.03 | 0.73-1.44 |
| Northern Ireland (ref: England) | 0.76 | 0.46-1.25 | 0.86 | 0.49-1.51 |
| *Quitting (ref: Occasional Smoking)* |  |  |  |  |
| Policy Implementation | 0.66 | 0.41-1.09 | 0.57 | 0.35-0.94 |
| Years Since Implementation | 0.85 | 0.67-1.08 | 0.99 | 0.75-1.32 |
| Historical Year | 1.30 | 1.15-1.47 | 1.26 | 1.11-1.43 |
| Historical Year^2^ | 1.02 | 1.01-1.03 | 1.01 | 1.00-1.02 |
| Other Qualifications (ref: degree) | 1.13 | 0.84-1.52 | 1.14 | 0.85-1.53 |
| No Qualifications (ref: degree) | 1.10 | 0.74-1.65 | 1.11 | 0.74-1.66 |
| Other Qualifications*Years Since Implementation | 1.01 | 0.94-1.08 | 1.00 | 0.94-1.08 |
| No Qualifications*Years Since Implementation | 0.99 | 0.91-1.09 | 0.99 | 0.90-1.09 |
| Tobacco Taxation | 1.00 | 0.98-1.01 | 0.99 | 0.98-1.01 |
| Adult E-cigarette Prevalence | 0.75 | 0.64-0.88 | 0.73 | 0.62-0.85 |
| Male (ref: Female) | 0.78 | 0.66-0.94 | 0.78 | 0.66-0.93 |
| Age at Experimentation | 0.90 | 0.83-0.98 | 0.90 | 0.83-0.98 |
| Years of Experimentation | 2.17 | 1.71-2.75 | 2.15 | 1.70-2.73 |
| Wales (ref: England) | 1.31 | 0.99-1.75 | 1.31 | 0.99-1.75 |
| Scotland (ref: England) | 1.49 | 1.14-1.95 | 1.36 | 1.06-1.76 |
| Northern Ireland (ref: England) | 1.15 | 0.79-1.68 | 1.12 | 0.72-1.74 |
|  |  |  |  |  |
