# Supplementary material for: Impact of UK Tobacco Control Policies on Inequalities in Youth Smoking Uptake: A Natural Experiment Study
Source: Nicotine Tob Res. 2020 May 29;22(11):1973–80. doi: 10.1093/ntr/ntaa101 (PMC7593354; doi:10.1093/ntr/ntaa101)
Supplement: ntaa101_suppl_Supplementary_Table_4 [file ntaa101_suppl_supplementary_table_4.docx]

**Supplementary Table 4: Odds Ratios Associated with Smoking Transitions –Combined Model**

|  | OR | 95% CIs |
| --- | --- | --- |
| *Initiation (ref: Never Smoked)* |  |  |
| Smoke-Free Legislation Implementation | 1.03 | 0.72-1.49 |
| Years Since Smoke-Free Legislation Implementation | 1.15 | 0.86-1.56 |
| Historical Year | 0.73 | 0.69-0.77 |
| Historical Year^2^ | 0.98 | 0.98-0.99 |
| Other Qualifications (ref: degree) | 1.47 | 1.29-1.67 |
| No Qualifications (ref: degree) | 2.05 | 1.73-2.43 |
| Other Qualifications*Years Since Smoke-Free Legislation Implementation | 0.93 | 0.69-1.27 |
| No Qualifications*Years Since Smoke-Free Legislation Implementation | 0.84 | 0.57-1.25 |
| Tobacco Taxation | 1.02 | 1.01-1.03 |
| Adult E-cigarette Prevalence | 1.06 | 1.00-1.13 |
| Male (ref: Female) | 0.93 | 0.87-1.00 |
| Age | 1.89 | 1.84-1.94 |
| Wales (ref: England) | 1.06 | 0.95-1.19 |
| Scotland (ref: England) | 1.04 | 0.90-1.19 |
| Northern Ireland (ref: England) | 1.06 | 0.86-1.30 |
| Change in Legal Age for Purchase Implementation | 0.63 | 0.43-0.92 |
| Years Since Change in Legal Age for Purchase Implementation | 1.03 | 0.75-1.41 |
| Other Qualifications*Years Since Change in Legal Age for Purchase Implementation | 1.06 | 0.78-1.45 |
| No Qualifications*Years Since Change in Legal Age for Purchase Implementation | 1.15 | 0.77-1.72 |
| *Experimentation (ref: Initiated only)* |  |  |
| Smoke-Free Legislation Implementation | 0.76 | 0.46-1.26 |
| Years Since Smoke-Free Legislation Implementation | 0.90 | 0.58-1.37 |
| Historical Year | 0.98 | 0.91-1.05 |
| Historical Year^2^ | 1.00 | 0.99-1.01 |
| Other Qualifications (ref: degree) | 1.02 | 0.83-1.24 |
| No Qualifications (ref: degree) | 1.58 | 1.22-2.06 |
| Other Qualifications*Years Since Smoke-Free Legislation Implementation | 0.99 | 0.61-1.59 |
| No Qualifications*Years Since Smoke-Free Legislation Implementation | 0.74 | 0.40-1.38 |
| Tobacco Taxation | 0.99 | 0.98-1.00 |
| Adult E-cigarette Prevalence | 0.95 | 0.87-1.05 |
| Male (ref: Female) | 0.93 | 0.84-1.02 |
| Age at Initiation | 1.18 | 1.12-1.23 |
| Years Since Initiation | 1.31 | 1.23-1.43 |
| Wales (ref: England) | 0.94 | 0.80-1.11 |
| Scotland (ref: England) | 1.00 | 0.82-1.22 |
| Northern Ireland (ref: England) | 1.15 | 0.85-1.55 |
| Change in Legal Age for Purchase Implementation | 1.35 | 0.79-2.31 |
| Years Since Change in Legal Age for Purchase Implementation | 1.37 | 0.86-2.19 |
| Other Qualifications*Years Since Change in Legal Age for Purchase Implementation | 1.02 | 0.63-1.66 |
| No Qualifications*Years Since Change in Legal Age for Purchase Implementation | 1.30 | 0.69-2.46 |
| *Escalation to Daily Smoking (ref: Occasional Smoking)* |  |  |
| Smoke-Free Legislation Implementation | 0.76 | 0.24-2.39 |
| Years Since Smoke-Free Legislation Implementation | 0.97 | 0.40-2.35 |
| Historical Year | 1.01 | 0.86-1.19 |
| Historical Year^2^ | 1.00 | 0.98-1.01 |
| Other Qualifications (ref: degree) | 1.43 | 0.98-2.08 |
| No Qualifications (ref: degree) | 1.90 | 1.19-3.04 |
| Other Qualifications*Years Since Smoke-Free Legislation Implementation | 1.31 | 0.51-3.36 |
| No Qualifications*Years Since Smoke-Free Legislation Implementation | 0.80 | 0.23-2.80 |
| Tobacco Taxation | 0.99 | 0.97-1.01 |
| Adult E-cigarette Prevalence | 0.83 | 0.68-1.02 |
| Male (ref: Female) | 0.79 | 0.65-0.96 |
| Age at Experimentation | 2.08 | 1.57-2.76 |
| Years of Experimentation | 1.29 | 1.18-1.42 |
| Wales (ref: England) | 1.20 | 0.85-1.70 |
| Scotland (ref: England) | 1.01 | 0.67-1.52 |
| Northern Ireland (ref: England) | 0.85 | 0.42-1.70 |
| Change in Legal Age for Purchase Implementation | 1.15 | 0.35-3.76 |
| Years Since Change in Legal Age for Purchase Implementation | 1.30 | 0.51-3.34 |
| Other Qualifications*Years Since Change in Legal Age for Purchase Implementation | 0.75 | 0.29-1.93 |
| No Qualifications*Years Since Change in Legal Age for Purchase Implementation | 1.25 | 0.35-4.45 |
| *Quitting (ref: Occasional Smoking)* |  |  |
| Smoke-Free Legislation Implementation | 1.07 | 0.44-2.58 |
| Years Since Smoke-Free Legislation Implementation | 0.57 | 0.27-1.20 |
| Historical Year | 1.26 | 1.11-1.44 |
| Historical Year^2^ | 1.01 | 1.00-1.02 |
| Other Qualifications (ref: degree) | 1.10 | 0.82-1.49 |
| No Qualifications (ref: degree) | 1.09 | 0.72-1.63 |
| Other Qualifications*Years Since Smoke-Free Legislation Implementation | 1.56 | 0.71-3.42 |
| No Qualifications*Years Since Smoke-Free Legislation Implementation | 1.09 | 0.39-3.03 |
| Tobacco Taxation | 0.99 | 0.98-1.01 |
| Adult E-cigarette Prevalence | 0.73 | 0.62-0.86 |
| Male (ref: Female) | 0.78 | 0.65-0.94 |
| Age at Experimentation | 0.90 | 0.83-0.98 |
| Years of Experimentation | 2.17 | 1.71-2.76 |
| Wales (ref: England) | 1.33 | 1.00-1.77 |
| Scotland (ref: England) | 1.55 | 1.11-2.17 |
| Northern Ireland (ref: England) | 1.34 | 0.74-2.42 |
| Change in Legal Age for Purchase Implementation | 0.59 | 0.24-1.49 |
| Years Since Change in Legal Age for Purchase Implementation | 1.72 | 0.79-3.78 |
| Other Qualifications*Years Since Change in Legal Age for Purchase Implementation | 0.64 | 0.29-1.42 |
| No Qualifications*Years Since Change in Legal Age for Purchase Implementation | 0.91 | 0.32-2.65 |
|  |  |  |
