# Supplementary material for: Impact of UK Tobacco Control Policies on Inequalities in Youth Smoking Uptake: A Natural Experiment Study
Source: Nicotine Tob Res. 2020 May 29;22(11):1973–80. doi: 10.1093/ntr/ntaa101 (PMC7593354; doi:10.1093/ntr/ntaa101)
Supplement: ntaa101_suppl_Supplementary_Table_5 [file ntaa101_suppl_supplementary_table_5.docx]

**Supplementary Table 5: Complete Case Analysis**

|  | **Smoke-Free Legislation** | | **Change in Legal Age for Purchase** | |
| --- | --- | --- | --- | --- |
|  | OR | 95% CIs | OR | 95% CIs |
| *Initiation (ref: Never Smoked)* |  |  |  |  |
| Policy Implementation | 0.69 | 0.70-1.27 | 1.03 | 0.76-1.40 |
| Years Since Implementation | 0.93 | 0.77-1.11 | 0.91 | 0.74-1.13 |
| Historical Year | 0.84 | 0.77-0.93 | 0.83 | 0.75-0.91 |
| Historical Year^2^ | 0.99 | 0.98-0.99 | 0.99 | 0.98-1.00 |
| Other Qualifications (ref: degree) | 1.38 | 1.14-1.67 | 1.37 | 1.13-1.65 |
| No Qualifications (ref: degree) | 2.37 | 1.81-3.11 | 2.35 | 1.80-3.08 |
| Other Qualifications*Years Since Implementation | 0.97 | 0.93-1.02 | 0.98 | 0.93-1.02 |
| No Qualifications*Years Since Implementation | 0.91 | 0.85-0.97 | 0.91 | 0.84-0.97 |
| Tobacco Taxation | 1.01 | 0.99-1.02 | 1.01 | 0.99-1.02 |
| Adult E-cigarette Prevalence | 1.18 | 1.03-1.34 | 1.21 | 1.06-1.38 |
| Male (ref: Female) | 0.88 | 0.78-0.98 | 0.88 | 0.78-0.98 |
| Age | 1.43 | 1.37-1.50 | 1.89 | 1.83-1.94 |
| Wales (ref: England) | 0.95 | 0.79-1.15 | 0.95 | 0.79-1.15 |
| Scotland (ref: England) | 1.24 | 1.04-1.49 | 1.19 | 1.00-1.41 |
| Northern Ireland (ref: England) | 1.03 | 0.75-1.42 | 0.95 | 0.66-1.36 |
| *Experimentation (ref: Initiated only)* |  |  |  |  |
| Policy Implementation | 0.95 | 0.61-1.49 | 0.85 | 0.54-1.35 |
| Years Since Implementation | 0.71 | 0.53-0.95 | 0.69 | 0.48-0.98 |
| Historical Year | 1.12 | 0.97-1.30 | 1.14 | 0.98-1.31 |
| Historical Year^2^ | 1.01 | 1.00-1.03 | 1.02 | 1.00-1.03 |
| Other Qualifications (ref: degree) | 1.02 | 0.77-1.36 | 1.03 | 0.78-1.36 |
| No Qualifications (ref: degree) | 1.49 | 0.99-2.23 | 1.51 | 1.01-2.25 |
| Other Qualifications*Years Since Implementation | 0.98 | 0.91-1.05 | 0.98 | 0.91-1.05 |
| No Qualifications*Years Since Implementation | 0.94 | 0.84-1.05 | 0.94 | 0.84-1.05 |
| Tobacco Taxation | 1.01 | 0.99-1.03 | 1.01 | 0.99-1.03 |
| Adult E-cigarette Prevalence | 1.04 | 0.83-1.31 | 1.04 | 0.83-1.32 |
| Male (ref: Female) | 0.83 | 0.70-1.12 | 0.83 | 0.70-0.99 |
| Age at Initiation | 1.04 | 0.96-1.12 | 1.03 | 0.96-1.12 |
| Years Since Initiation | 0.94 | 0.84-1.05 | 0.94 | 0.84-1.04 |
| Wales (ref: England) | 0.77 | 0.57-1.02 | 0.76 | 0.57-1.02 |
| Scotland (ref: England) | 0.75 | 0.57-0.99 | 0.66 | 0.51-0.85 |
| Northern Ireland (ref: England) | 0.52 | 0.30-0.92 | 0.39 | 0.21-0.73 |
| *Escalation to Daily Smoking (ref: Occasional Smoking)* |  |  |  |  |
| Policy Implementation | 0.87 | 0.36-2.11 | 0.79 | 0.32-1.92 |
| Years Since Implementation | 0.86 | 0.48-1.56 | 0.74 | 0.38-1.45 |
| Historical Year | 1.12 | 0.84-1.49 | 1.15 | 0.87-1.53 |
| Historical Year^2^ | 1.01 | 0.98-1.03 | 1.01 | 0.99-1.04 |
| Other Qualifications (ref: degree) | 1.63 | 0.96-2.76 | 1.64 | 0.98-2.76 |
| No Qualifications (ref: degree) | 2.11 | 0.99-4.50 | 2.09 | 0.99-4.42 |
| Other Qualifications*Years Since Implementation | 0.91 | 0.78-1.05 | 0.90 | 0.78-1.04 |
| No Qualifications*Years Since Implementation | 1.00 | 0.81-1.22 | 1.00 | 0.81-1.23 |
| Tobacco Taxation | 1.01 | 0.97-1.06 | 1.02 | 0.98-1.07 |
| Adult E-cigarette Prevalence | 0.68 | 0.40-1.14 | 0.67 | 0.40-1.14 |
| Male (ref: Female) | 0.64 | 0.40-1.14 | 0.64 | 0.40-1.14 |
| Age at Experimentation | 1.20 | 1.03-1.39 | 1.19 | 1.03-1.38 |
| Years of Experimentation | 3.05 | 2.03-4.58 | 3.02 | 2.01-4.54 |
| Wales (ref: England) | 0.92 | 0.51-1.66 | 0.92 | 0.51-1.66 |
| Scotland (ref: England) | 0.80 | 0.44-1.45 | 0.73 | 0.41-1.29 |
| Northern Ireland (ref: England) | 0.83 | 0.24-2.95 | 0.63 | 0.16-2.48 |
| *Quitting (ref: Occasional Smoking)* |  |  |  |  |
| Policy Implementation | 1.13 | 0.52-2.45 | 0.56 | 0.25-1.25 |
| Years Since Implementation | 0.57 | 0.33-0.96 | 0.72 | 0.39-1.31 |
| Historical Year | 1.09 | 0.85-1.40 | 1.18 | 0.93-1.51 |
| Historical Year^2^ | 1.02 | 0.99-1.04 | 1.02 | 0.99-1.04 |
| Other Qualifications (ref: degree) | 1.45 | 0.90-2.34 | 1.50 | 0.93-2.41 |
| No Qualifications (ref: degree) | 1.78 | 0.90-3.50 | 1.82 | 0.93-3.57 |
| Other Qualifications*Years Since Implementation | 1.06 | 0.94-1.21 | 1.05 | 0.93-1.20 |
| No Qualifications*Years Since Implementation | 1.03 | 0.84-1.25 | 1.01 | 0.82-1.24 |
| Tobacco Taxation | 1.05 | 1.01-1.08 | 1.03 | 0.99-1.07 |
| Adult E-cigarette Prevalence | 0.65 | 0.42-0.99 | 0.54 | 0.35-0.83 |
| Male (ref: Female) | 0.83 | 0.61-1.13 | 0.84 | 0.62-1.14 |
| Age at Experimentation | 0.83 | 0.73-0.94 | 0.83 | 0.73-0.94 |
| Years of Experimentation | 2.79 | 1.93-4.04 | 2.79 | 1.93-4.03 |
| Wales (ref: England) | 1.35 | 0.81-2.24 | 1.31 | 0.79-2.18 |
| Scotland (ref: England) | 1.52 | 0.93-2.47 | 1.33 | 0.84-2.12 |
| Northern Ireland (ref: England) | 1.03 | 0.37-2.87 | 0.83 | 0.27-2.55 |
|  |  |  |  |  |
